# Concurrent validity of human pose tracking in video for measuring gait parameters in older adults: a preliminary analysis with multiple trackers, viewing angles, and walking directions

**DOI:** 10.1186/s12984-021-00933-0

**Published:** 2021-09-15

**Authors:** Sina Mehdizadeh, Hoda Nabavi, Andrea Sabo, Twinkle Arora, Andrea Iaboni, Babak Taati

**Affiliations:** 1 Department of Psychiatry, University of Toronto, Toronto, ON Canada; 2 Institute of Biomaterials and Biomedical Engineering, University of Toronto, Toronto, ON Canada; 3 Department of Computer Science, University of Toronto, Toronto, ON Canada; 4 KITE- Toronto Rehabilitation Institute, University Health Network, 550 University Ave., Toronto, ON M5G 2A2 Canada; 5 Centre for Mental Health, University Health Network, Toronto, ON Canada; 6 Vector Institute for Artificial Intelligence, Toronto, ON Canada

**Keywords:** Walking, Human pose estimation, Deep learning, Gait

## Abstract

**Background:**

Many of the available gait monitoring technologies are expensive, require specialized expertise, are time consuming to use, and are not widely available for clinical use. The advent of video-based pose tracking provides an opportunity for inexpensive automated analysis of human walking in older adults using video cameras. However, there is a need to validate gait parameters calculated by these algorithms against gold standard methods for measuring human gait data in this population.

**Methods:**

We compared quantitative gait variables of 11 older adults (mean age = 85.2) calculated from video recordings using three pose trackers (AlphaPose, OpenPose, Detectron) to those calculated from a 3D motion capture system. We performed comparisons for videos captured by two cameras at two different viewing angles, and viewed from the front or back. We also analyzed the data when including gait variables of individual steps of each participant or each participant’s averaged gait variables.

**Results:**

Our findings revealed that, i) temporal (cadence and step time), but not spatial and variability gait measures (step width, estimated margin of stability, coefficient of variation of step time and width), calculated from the video pose tracking algorithms correlate significantly to that of motion capture system, and ii) there are minimal differences between the two camera heights, and walks viewed from the front or back in terms of correlation of gait variables, and iii) gait variables extracted from AlphaPose and Detectron had the highest agreement while OpenPose had the lowest agreement.

**Conclusions:**

There are important opportunities to evaluate models capable of 3D pose estimation in video data, improve the training of pose-tracking algorithms for older adult and clinical populations, and develop video-based 3D pose trackers specifically optimized for quantitative gait measurement.

## Background

Clinically established techniques for examining gait quality in older adults typically require technologies such as motion capture systems which are expensive and time consuming, require specialized expertise and staff to operate, and are not widely available for clinical use. As a result, gait monitoring practices have mainly involved cross-sectional gait assessments in laboratory settings or under experimental conditions which do not reflect the cognitive and physical demands of natural walking or usual locomotion [1].

With the advent of commercially available depth cameras, specifically the Kinect sensor (Microsoft, Redmond, WA), researchers were able to monitor natural walking of participants [2–7]. However, the Kinect camera has a limited depth of field (0.5 to 4.5 m) which can only capture few steps. This limitation, along with concerns about cost and potential hardware obsolescence (the sensor was commercially unavailable for an extended period until a newer version was released) motivate adopting other technologies for the purpose of natural gait monitoring. Although other depth sensing cameras are available, it would be ideal if technologies can make use of regular videos from cameras that are ubiquitous, such as surveillance cameras.

Advances in computer vision technology and human pose estimation in image/video data can address these limitations. A number of algorithms have been developed for human pose tracking that are capable of automated analysis of human walking using only standard RGB camera videos [8–15]. These algorithms use deep learning models that are trained on a large corpus of annotated videos, resulting in models capable of detecting body segments (head, hands, knees, feet, etc.) in new videos outside of the training dataset. These packages are freely available and can be used to process videos of human walking in any setting with minimal cost and technical expertise [15]. Gait parameters can subsequently be computed from the sequence of tracked body parts [16]. However, for use in clinical applications, there is a need to validate gait variables calculated from pose tracking data against gold standard methods for measuring human gait data, e.g., three-dimensional (3D) motion capture systems [15].

Previous studies on the validation of video pose tracking algorithms mainly used a single pose tracking algorithm, mainly OpenPose [8], in sagittal view, and healthy young adults [9, 11, 12, 15, 17]. Less is known about the performance of other publicly available pose tracking algorithms such as AlphaPose [13] or Detectron [14] particularly for pose tracking of gait in a frontal view and in older adult populations. There are several reasons that this analysis is valuable and necessary: i) comparison of different pose trackers allows researchers to choose the most appropriate one for the purpose, ii) recording walks in a frontal view allows the capture of more steps and an analysis of stability in the frontal plane, and iii) pose tracking algorithms require validation in older adults as their posture and gait are different to that in young adults and is characterized by lower speed, and greater variability [18].

The aim of this study was, therefore, to investigate the concurrent validity of spatiotemporal gait measurement in the frontal plane based on three common pose trackers (AlphaPose [13], OpenPose [8], and Detectron [14]) against a 3D motion capture system by doing a correlation analysis between the gait variables calculated from the two systems in older adults.

## Methods

### Participants

Participants were residents (older adults age > 65 years) of a retirement home who were able to provide consent to participate in the study. The University of Toronto Ethics Board approved the study protocol. Residents of the retirement home were sent a recruitment letter briefly describing the study, and expressed their interest in participation by calling the research assistant. Participants provided written consent before participating in the study. The inclusion criteria were being older than 65 years and an ability to walk independently over a distance of 20 m. There were no exclusion criteria. Participants declared that did not have any pain or fatigue that could affect their walking at the time of experiments. The following clinical tests were performed by the research assistant: the Mini-Mental State Examination [19], the Tinneti performance-oriented mobility assessment (POMA) for balance and gait [20], the Berg balance scale [21], and timed-up-and-go [22].

### Task

Participants walked back and forth for one minute along the long axis of large room. The walking distance was approximately 13 m and the walking surface was flat. Participants were instructed to walk at their normal pace with the ‘go’ signal from the assessor and stopped walking with the ‘stop’ signal. A clinical assistant walked beside the participants at a safe distance but did not provide any pacing or physical support.

### Motion capture system

An Xsens MVN Awinda system (Xsens, Enschede, Netherlands) comprising of seven wireless inertial measurement units (IMUs), a receiver hub and straps was used to record participants’ walking at 100 Hz. The seven lower body IMU sensors were attached to the right and left feet, shanks, thighs and the sacrum. The participants’ height was measured and the sensors were calibrated before starting the walking task. The MVN Analyze software (Xsens, Enschede, Netherlands) was used to record the walking tasks. The Xsens IMU system is valid and reliable 3D motion capture system with the accuracy comparable to traditional optical motion capture systems [23, 24] with the added benefit of not being constrained to the lab space, which was required for this project. In particular, the concurrent validity of the Xsens system compared to 3D marker-based motion capture was reported to be greater than 0.8 for the sagittal and frontal joint angles [23, 24]. Teufl, Lorenz [25] also reported that gait event detection accuracy of the Xsens system was about 99% with a relative root mean square error of 0.90–4.40% for most gait variables.

### Cameras

Two Motorola Moto G5 Play cell phones (Motorola, Chicago, IL), equipped with a 13 mega pixel camera capable of recording videos at 30 frame per second at 1080p resolution, were used to record the walking videos. These cameras were placed at two different heights of 111 cm (approximately eye-level) and 205 cm, chosen to mimic wall-mounted (straight) and ceiling-mounted (tilted down) camera viewing angles.

### Video pose tracking

The recorded videos were first cropped temporally, selecting only the sections of the recordings where the participant was continuously walking towards (front view) or away (back view) from the cameras. The sections of the recordings where the participant was turning were excluded from analysis because the pose tracking algorithms could not accurately estimate body landmarks during the turns. Open-source human pose estimation libraries were then used to extract the joint positions in each frame of the cropped videos. The three libraries investigated were OpenPose [8](Windows demo release 1.5.1), Detectron [14] (R-CNN R101-FPN pretrained model backbone), and AlphaPose [13] (YOLOv3-spp detector, pretrained ResNet-50 backbone). All these three predict the location of key joints independently for each frame of an input video. OpenPose uses a bottom-up approach that first predicts part affinity fields (PAFs) and confidence maps of joints. The PAFs are subsequently used to perform part association and prediction of overall body poses. Conversely, Detectron and AlphaPose both implement top-down designs. Detectron employs an architecture that simultaneously predicts bounding boxes, segments, and joint keypoints. AlphaPose uses a sequential architecture that first places bounding boxes around each person in the image and then performs keypoint prediction within the bounding box.

The pose estimation models provide the lateral and vertical positions (in the frontal view) of body joints in each frame of the input video, as well as a score representing the model’s confidence in its prediction of the joint position. The predicted joint positions in each frame were aggregated temporally to obtain joint trajectories of the participant’s movement in each video. The confidence scores provided by the pose estimation libraries were used to identify and remove and linearly interpolate joint positions at time steps where the pose estimation library predicted a joint with low confidence. As the confidence scores output from the different pose estimation libraries are not calibrated, the threshold used to denote low confidence varied for each library (0.3 for OpenPose, 0.5 for AlphaPose, and 0.15 for Detectron). These threshold values were chosen by trial and error to make sure that the keypoints in less than 10 percent of the frames were missing or had low confidence. As also noted by Stenum,  et al. [15], the pose estimation libraries may sometimes erroneously label a joint on the left side of the body as the corresponding joint on the right side. To address this, the joint trajectories were visually inspected and these errors where manually corrected. Finally, a zero-lag second-order low-pass Butterworth filter with a cut-off frequency of 8 Hz was used to temporally smooth the joint positions [26]. Note that no synchronization between the two systems were required for the purpose of this study as we compare gait parameters calculated from the two systems and not the pose estimation at each time frame. However, we ensured that the first and last steps of each walking bout (front or back view) were the same to make sure that step-wise gait measures were calculated from the same sequence of steps.

### Gait variables

Gait variables were calculated for the recorded gait from the two systems, i.e. i) using the 3D joint coordinates extracted using the MVN Analyze (Xsens, Enschede, Netherlands), and ii) by processing the recorded color videos via the pose tracking algorithms to obtain 2D joint (pixel) coordinate. Spatial gait variables (e.g. step width) calculated from the video were normalized by hip width to account for the perspective. Gait variables used were cadence (number of steps per minutes), step time (the time between two consecutive foot strikes) and step width (the distance medial distance between the left and right feet at the time of foot strike) and their variability (coefficient of variation, CV, defined as the standard deviation divided by the mean), and estimated margin of stability (eMOS). The eMOS is the distance between velocity–corrected centre of mass from the foot at stance in the lateral direction). The eMOS is based on the margin of stability measure introduced initially by Hof [27]. It is calculated using Eq. (1):

1 $$eMOS = XCOM - BOS$$

where *XCOM* is called the extrapolated centre of mass and is calculated as $XCOM=COM+{V}_{COM}/\omega$**.** Here, the $COM$ is the position of the body centre of mass (sacrum in our study) in the mediolateral direction, ${V}_{COM}$ is the velocity of the centre of mass in the medio-lateral direction, and $\omega$ is the frequency of the leg (distance from scrum to the foot in our study) oscillation and is determined as $\omega =\sqrt{g/l}$ where $g$ is the gravity and $l$ is the leg length. The $BOS$ in Eq. (1) is the lateral boundary of the base of support which, in our study, is the lateral position of the foot when in stance. Due to the necessary adjustments made to Hof [27]’s measure of margin of stability, we named it *estimated* margin of stability (eMOS). These gait variables have been shown to be correlated with fall risk in the literature [28, 29]. The details of calculating these variables are presented in our previous papers [3, 6, 16, 30]. To calculate these gait variables, the steps in each walking bout were first identified. For the Xsens data, this was provided by the MVN Analyze software (Xsens, Enschede, Netherlands). For time series from the videos, this was done by detrending the vertical position of the ankle, and finding the local extrema and were compared to those provided by the IMU system (considering the different recording sampling rates) for all pose tracking algorithms and corrections were made when necessary. Gait variables for front view and back view walks were analyzed separately. Custom codes written in Matlab version R2018a (Mathworks, Natick, MA) was used to calculate all gait variables.

### Statistical analysis

Pearson’s correlation coefficients (R) were used to determine the correlation between the gait variables calculated from the video and from the inertial motion capture system as the gold standard. We compared these two sets of gait variables in 24 different conditions: 3 pose tracking algorithms (AlphaPose, OpenPose, and Detectron), 2 camera heights (eye-level and top), 2 walking views (front and back), and 2 calculation methods (individual steps and mean values of all steps over a walking bout). “Individual steps” refer to including gait variables of each individual step in the correlation analysis while “mean of all steps” refers to averaging gait variables over all of the steps of each walking bout of each participant. The correlation coefficients (R) and p-values were reported. The significance level was 0.05.

While we were not able to measure and compare the accuracy of the three vision-based pose tracking algorithms against our IMU system due to having different units (i.e. the gait measures from the IMU system are in meters while they are unitless in the pose tracking algorithms), we compared the performance of the three pose tracking algorithms with respect to each other by calculating (i) precision—the dispersion around the mean using coefficient of variation (CV), and (ii) the degrees of agreement between pairs of the pose tracking algorithms using Bland–Altman plots. Because the gait events calculated from the three pose trackers were similar to each other, Bland–Altman agreement plots were the same for the temporal gait variables (cadence and step time) calculated for the three trackers. We have thus presented the results of the Bland–Altman analysis for the spatial gait variables only (i.e. step width and eMOS).

## Results

**Fig. 1:**
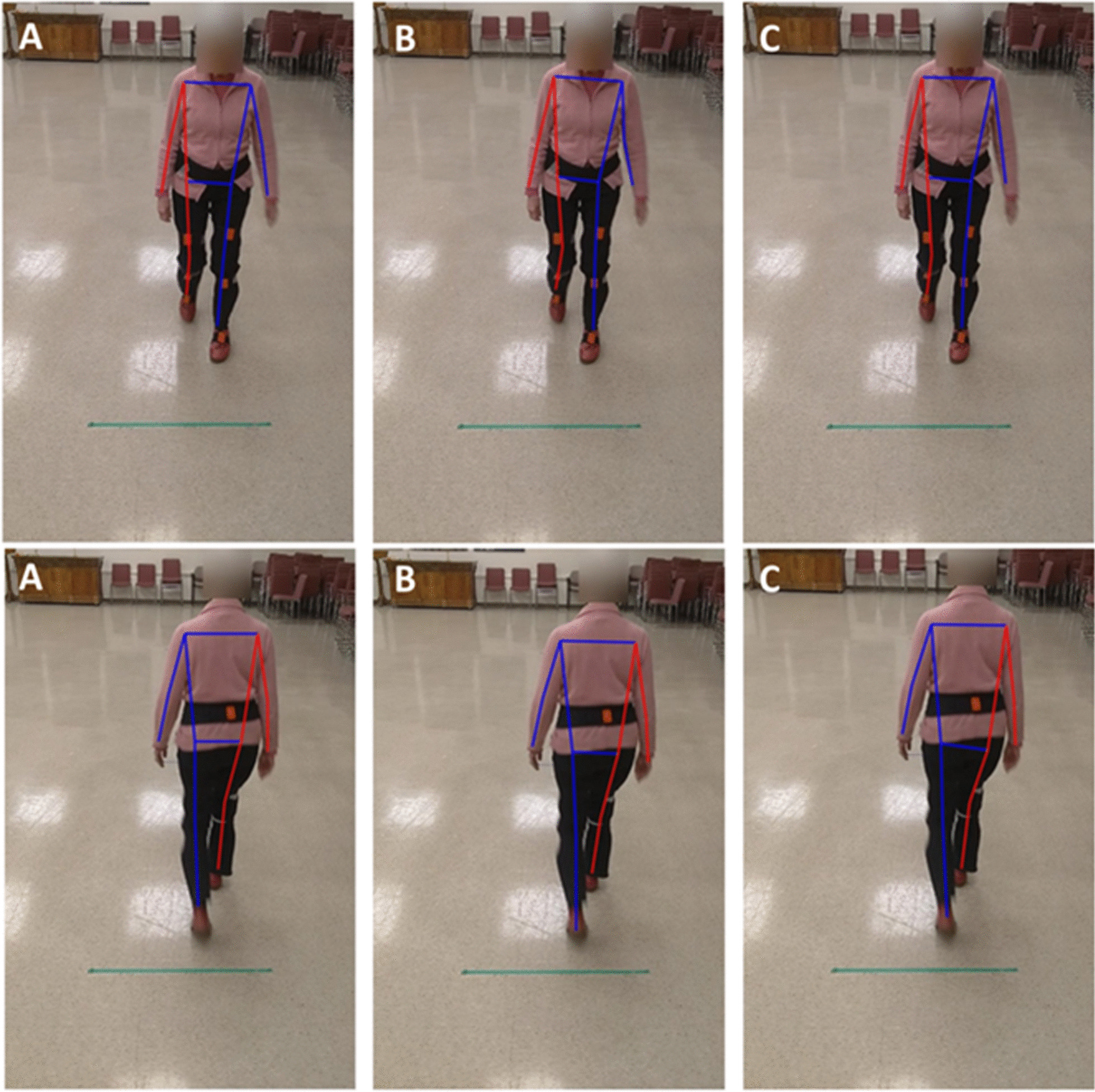
The pose tracking skeletons overlaid on the eye-level camera video for front (top row) and back (bottom row) view walks, **A** AlphaPose, **B** OpenPose, **C** Detectron

**Fig. 2:**
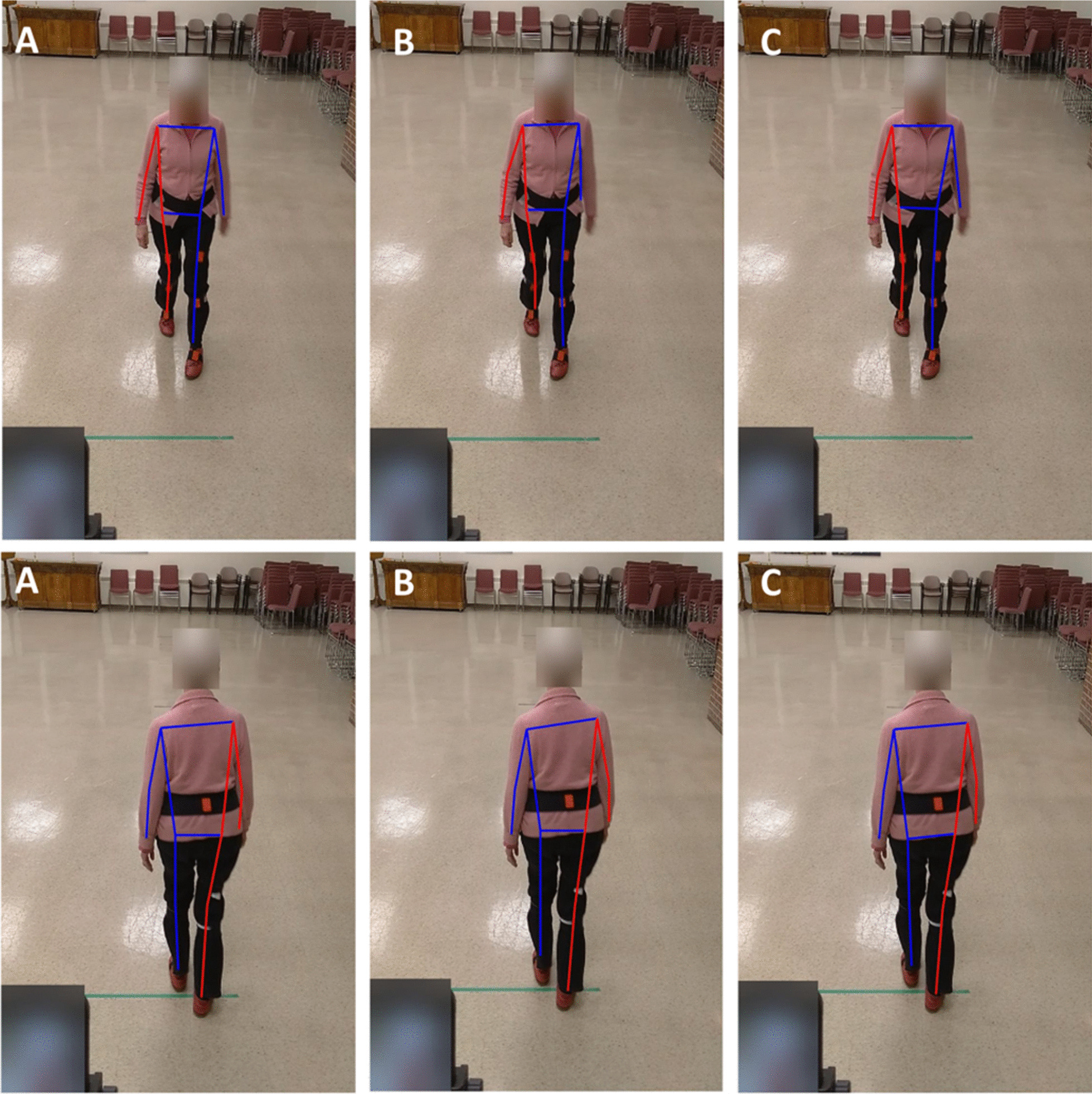
The pose tracking skeletons overlaid on the top camera video for front (top row) and back (bottom row) view walks, **A** AlphaPose, **B** OpenPose, **C** Detectron

**Fig. 3:**
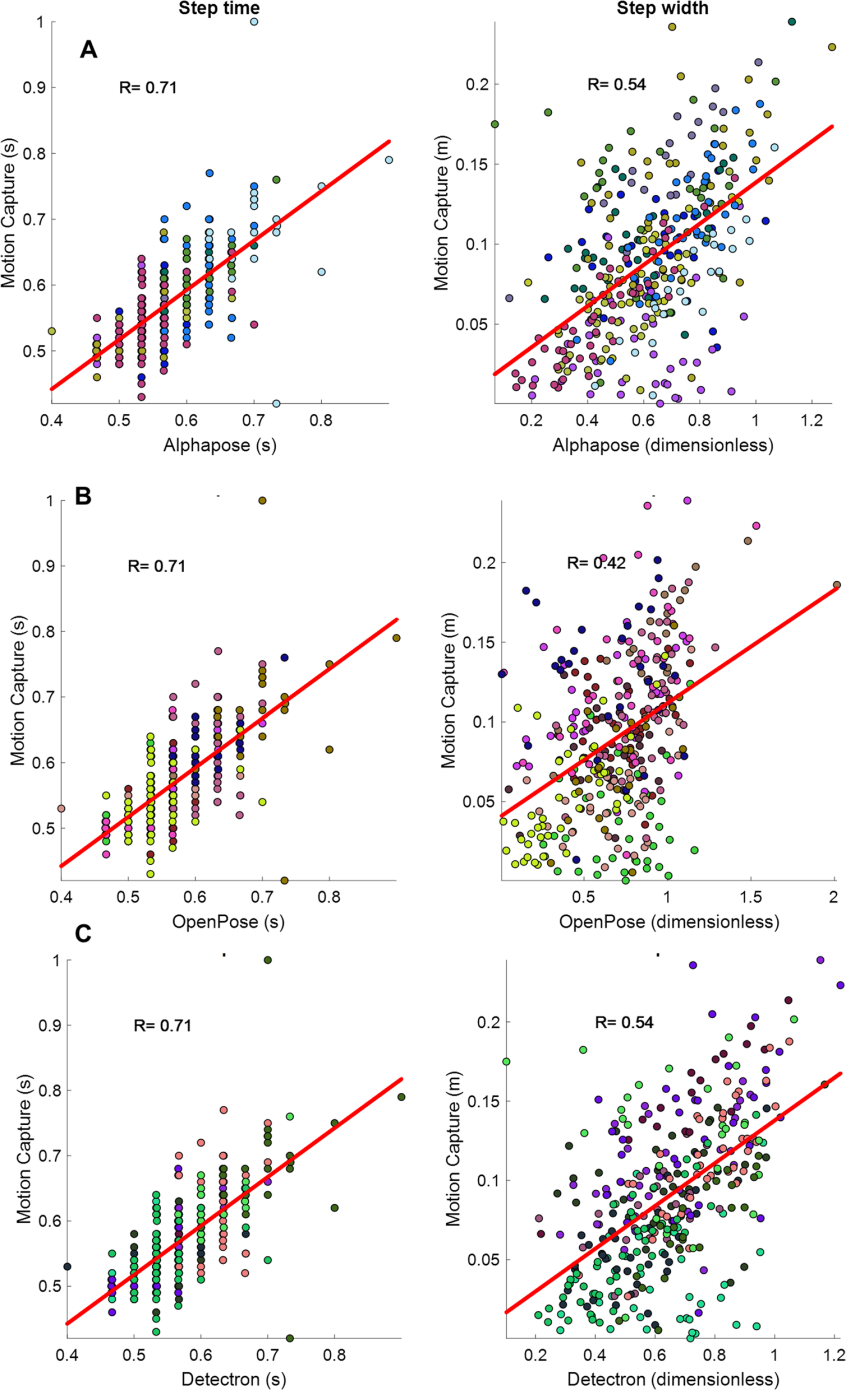
Scatter plots for one temporal (step time, left column), and one spatial (step width, right column) gait variable, including each individual step captured in the front view walking bout with the eye-level camera, **A** AlphaPose, **B** OpenPose, **C** Detectron. The colors are associated with different participants’ data. The thick red line is the fitted line. The correlation values are also shown in the figures

While the initial plan was to recruit 20 participants, the experiments were discontinued after recruiting 14 participants due to the Covid-19 pandemic. Out of the 14 participants who took part in the study, data for three were not suitable for analysis (their motion capture data had calibration problems) and thus discarded, leaving the data of 11 participants for analysis (mean age = 85.2). The demographics of the participants are presented in Table 1. In total, participants had 26 front and 20 back view walks, an average of 2.3 and 1.8 walking bouts per participant for front and back view walks, respectively. In addition, participants had a total of 354 steps in the front view walk and 244 in back view walk, equal to average of 32.2 and 22.2 steps per participant for front and back view walks, respectively. These equal to, on average, 14 (32.2/2.3) and 12.3 (22.2/1.8) steps per each front and back view walks, respectively. The average values of the six gait measures for the motion capture (Xsens) and the three pose tracking algorithms are presented in Table 2. A snapshot of the three pose tracking algorithms overlaid on one frame of the videos is presented in Figs. 1 and 2. An example of scatter plots for one temporal (step time), and one spatial (step width) gait variable, for the eye-level camera videos is presented in Fig. 3.

**Table 1:** Demographic results of the participants

Age (mean years ± SD)	85.2 ± 5.6
Number of men (%)	2 (18.1)
Weight (mean kg ± SD)	63.2 ± 12.2
Height (mean cm ± SD)	163.6 ± 9.7
Number of falls in past 6 months (%)	0 (0)
POMA-balance	12.8 ± 1.6
POMA-gait	11.7 ± 0.6
TUG (s)	12.2 ± 4.2
BBS	45.0 ± 6.3
MMSE	28.09 ± 3.5

*SD* standard deviation, *POMA* Tinneti performance-oriented mobility assessment, *TUG* time up-and-go, *BBS* Berg balance scale, *MMSE* Mini-Mental State Examination

**Table 2:** Average (standard deviation) of the gait variables calculated for the motion capture and video data using the three pose tracking algorithms

Gait variable	Motion capture		AlphaPose				OpenPose				Detectron			
	Front view walks	Back view walks	Front view walks		Back view walks		Front view walks		Back view walks		Front view walks		Back view walks	
			Top camera	Eye-level camera	Top camera	Eye-level camera	Top camera	Eye-level camera	Top camera	Eye-level camera	Top camera	Eye-level camera	Top camera	Eye-level camera
Cadence (steps per minute)	106.44 (8.10)	105.30 (8.98)	106.23 (9.14)	106.51 (8.62)	105.63 (9.39)	105.26 (9.30)	106.22 (9.14)	106.51 (8.62)	105.63 (9.40)	105.26 (9.29)	106.23 (9.14)	106.52 (8.62)	105.63 (9.39)	105.26 (9.29)
Step time (s)	0.57 (0.06)	0.58 (0.07)	0.57 (0.10)	0.57 (0.06)	0.58 (0.17)	0.58 (0.07)	0.57 (0.10)	0.57 (0.06)	0.58 (0.17)	0.58 (0.07)	0.57 (0.10)	0.57 (0.06)	0.58 (0.17)	0.58 (0.07)
Step width (m)*	0.09 (0.05)	0.10 (0.04)	0.63 (0.20)	0.62 (0.20)	0.55 (0.16)	0.54 (0.16)	0.62 (0.46)	0.67 (0.29)	0.59 (0.23)	0.54 (0.22)	0.66 (0.19)	0.64 (0.19)	0.86 (1.92)	0.54 (0.15)
CV step time	0.06 (0.03)	0.06 (0.04)	0.13 (0.10)	0.06 (0.02)	0.16 (0.18)	0.06 (0.03)	0.13 (0.10)	0.06 (0.02)	0.16 (0.18)	0.06 (0.03)	0.13 (0.10)	0.06 (0.02)	0.16 (0.18)	0.06 (0.03)
CV step width	0.42 (0.21)	0.37 (0.16)	0.27 (0.08)	0.30 (0.07)	0.27 (0.08)	0.28 (0.07)	0.38 (0.21)	0.38 (0.12)	0.37 (0.10)	0.39 (0.09)	0.25 (0.07)	0.28 (0.06)	0.29 (0.12)	0.27 (0.08)
eMOS (m)^a^	0.04 (0.02)	0.04 (0.02)	0.25 (0.12)	0.23 (0.10)	0.22 (0.09)	0.21 (0.08)	0.33 (0.14)	0.30 (0.14)	0.28 (0.12)	0.25 (0.11)	0.25 (0.12)	0.23 (0.09)	0.40 (0.15)	0.21 (0.09)

^a^Note that except for cadence and step time, the values calculated from the pose tracking algorithms are dimensionless (normalized by hip width) and cannot be directly compared with motion capture values

The pattern of correlations was similar between the three pose tracking algorithms and the motion capture and there were no major differences amongst the three algorithms. That is, for all three algorithms, the temporal gait measures (cadence and step time) had high (R > 0.7) to very high (R > 0.9) correlations to the motion capture values in most conditions, while the spatial measures mainly had low (R < 0.5) or moderate (0.5 < R < 0.7) correlations to the motion capture. In addition, averaging the gait measures over the steps of each participant (mean values in the tables) improved the correlations, but spatial gait measures remained non significant (p > 0.05), possibly because the number of samples also reduced by a large factor because of averaging. The details for all pose tracking algorithms are provided below.

### AlphaPose

When considering all individual steps (Table 3), cadence had the correlation of 0.99 (p < 0.001) and 1.00 (p < 0.001) for the front and back view walks of the eye-level camera, respectively. These values were 0.97 (p < 0.001) for both front and back view of the top cameras. In addition, step time had a correlation of 0.71 (p < 0.001) with the motion capture for both front and back view walks of the eye-level camera. All other correlations were low (R < 0.5) according to classification of correlation values suggested by [31]. Regarding eMOS results, the values of front view (R = 0.45, p < 0.001 for both cameras) walks was higher than back view walks (R = 0.14, p = 0.03 for eye-level camera and R = 0, p = 0.95 for top camera) in both eye-level and top cameras although the correlation were poor (R < 0.5) in all cases.

**Table 3:** Results of correlation analysis between gait variables calculated from the motion capture and from the AlphaPose pose tracking

Gait variable	Eye-level camera				Top camera			
	R	p	R	p	R	p	R	p
	Front view walks		Back view walks		Front view walks		Back view walks	
	Individual steps				Individual steps			
Cadence	0.99	< 0.001	1.00	< 0.001	0.97	< 0.001	0.97	< 0.001
Step time	0.71	< 0.001	0.71	< 0.001	0.48	< 0.001	0.31	< 0.001
Step width	0.54	< 0.001	0.31	< 0.001	0.62	< 0.001	0.25	< 0.001
Step time CV	0.17	0.40	− 0.05	0.85	0.03	0.87	0.30	0.20
Step width CV	0.45	0.02	0.36	0.12	0.36	0.07	0.30	0.20
eMOS	0.45	< 0.001	0.14	0.03	0.45	< 0.001	0	0.95

Gait variable	Eye-level camera				Top camera			
	R	p	R	p	R	p	R	p
	Front view walks		Back view walks		Front view walks		Back view walks	
	Mean of all steps				Mean of all steps			
Cadence	1.00	< 0.001	1.00	< 0.001	0.99	< 0.001	0.98	< 0.001
Step time	1.00	< 0.001	1.00	< 0.001	0.99	< 0.001	0.99	< 0.001
Step width	0.57	0.07	0.42	0.20	0.66	0.03	0.58	0.06
Step time CV	0.52	0.10	0.22	0.51	0.13	0.71	0.50	0.12
Step width CV	0.38	0.25	0.41	0.21	0.33	0.33	0.23	0.50
eMOS	0.61	0.11	0.66	0.04	0.58	0.13	0.60	0.09

Calculating the correlations for averaged gait variables (mean values in Table 3) increased the correlation values. However, note that although several gait variables– front view step width (R = 0.57, p = 0.07) in the eye-level camera and step width (R = 0.58, p = 0.06) and eMOS of back view walk (R = 0.60, p = 0.09) in the top camera – had a moderate correlation (R > 0.5) with nonsignificant p-values.

### OpenPose

When including all individual steps (Table 4), cadence had the correlation of 0.99 (p < 0.001) and 1.00 (p < 0.001) for both front and back view walks of the eye-level camera, respectively. These values were 0.97 (p < 0.001) for both front and back view of the top cameras.

**Table 4:** Results of correlation analysis between gait variables calculated from the motion capture and from the OpenPose pose tracking

Gait variable	Eye-level camera				Top camera			
	R	p	R	p	R	p	R	p
	Front view walks		Back view walks		Front view walks		Back view walks	
	Individual steps				Individual steps			
Cadence	0.99	< 0.001	1.00	< 0.001	0.97	< 0.001	0.97	< 0.001
Step time	0.71	< 0.001	0.71	< 0.001	0.48	< 0.001	0.31	< 0.001
Step width	0.42	< 0.001	0.34	< 0.001	0.44	< 0.001	0.17	0.01
Step time CV	0.17	0.40	− 0.05	0.85	0.03	0.87	0.30	0.20
Step width CV	0.27	0.18	0.60	< 0.001	− 0.16	0.43	0.27	0.26
eMOS	0.30	< 0.001	0.09	0.17	0.27	< 0.001	0.03	0.68

Gait variable	Eye-level camera				Top camera			
	R	p	R	p	R	p	R	p
	Front view walks		Back view walks		Front view walks		Back view walks	
	Mean of all steps				Mean of all steps			
Cadence	1.00	< 0.001	1.00	< 0.001	0.99	< 0.001	0.98	< 0.001
Step time	1.00	< 0.001	1.00	< 0.001	0.99	< 0.001	0.99	< 0.001
Step width	0.56	0.07	0.54	0.09	0.55	0.08	0.39	0.24
Step time CV	0.52	0.10	0.22	0.51	0.13	0.71	0.50	0.12
Step width CV	0.18	0.61	0.60	0.05	− 0.06	0.87	0.15	0.65
eMOS	0.17	0.78	0.79	0.02	− 0.25	0.63	0.30	0.43

Similar to AlphaPose, here also averaging the gait variables over all steps for each participant increased the correlation values. Cadence and step time had the correlation of 1.00 (p < 0.001) for eye-level and 0.99 (p < 0.001) for top cameras in both front and back view walks, and MOS of the back view walk in eye-level camera had the correlation of 0.79 (p = 0.02). Variables with moderate correlations included step widths (R = 0.56, p = 0.07, and R = 0.54, p = 0.09 for the front and back view walks, respectively), and back view walk step width CV (R = 0.60, p = 0.05) in the eye-level camera and front view walk step width (R = 0.55, p = 0.08) in the top camera.

### Detectron

Cadence had a correlation of 0.99 (front view) and 1.00 (back view) in the eye-level camera (p < 0.001) and 0.97 (in both front and back views) in the top camera using the Detectron pose tracking algorithm (Table 5). Step time’s correlation was 0.71 (p < 0.001) for the eye-level camera, step width of front view walk had a correlation of 0.54 (p < 0.001) for the eye-level camera and 0.64 (p < 0.001) for the top camera, and the correlation was 0.51 (p = 0.02) for the step width CV of the back view walk in the eye-level camera.

**Table 5:** Results of correlation analysis between gait variables calculated from the motion capture and from the Detectron pose tracking

Gait variable	Eye-level camera				Top camera			
	R	p	R	p	R	p	R	p
	Front view walks		Back view walks		Front view walks		Back view walks	
	Individual steps				Individual steps			
Cadence	0.99	< 0.001	1.00	< 0.001	0.97	< 0.001	0.97	< 0.001
Step time	0.71	< 0.001	0.71	< 0.001	0.48	< 0.001	0.31	< 0.001
Step width	0.54	< 0.001	0.38	< 0.001	0.64	< 0.001	0.19	< 0.001
Step time CV	0.16	0.43	− 0.05	0.85	0.03	0.87	0.30	0.20
Step width CV	0.36	0.07	0.51	0.02	0.32	0.11	0.07	0.76
eMOS	0.44	< 0.001	0.12	0.05	0.44	< 0.001	0.01	0.83

Gait variable	Eye-level camera				Top camera			
	R	p	R	p	R	p	R	p
	Front view walks		Back view walks		Front view walks		Back view walks	
	Mean of all steps				Mean of all steps			
Cadence	1.00	< 0.001	1.00	< 0.001	0.99	< 0.001	0.98	< 0.001
Step time	1.00	< 0.001	1.00	< 0.001	0.99	< 0.001	0.99	< 0.001
Step width	0.66	0.03	0.51	0.11	0.74	0.01	0.53	0.09
Step time CV	0.53	0.09	0.22	0.51	0.13	0.71	0.50	0.12
Step width CV	0.25	0.46	0.48	0.13	0.31	0.35	0.24	0.47
eMOS	0.61	0.11	0.76	0.01	0.59	0.12	0.33	0.35

Similar to other two pose tracking algorithms, averaging the gait variables over all steps for each participant increased the correlation values. That is, cadence and step time had a correlation of 1.00 (p < 0.001) for the eye-level camera and 0.99 (p < 0.001) for top camera. The correlation for the eMOS of back view walk in the eye-level camera was 0.76 (p = 0.01) while this value was 0.74 (p = 0.01) for the step width of front view walk in the top camera. Other moderate correlations include step time CV of front view walk in the eye-level camera (R = 0.53, p = 0.09) and step width of the back view walk in the top camera (R = 0.53, p = 0.09) although their p-values were not significant.

### Comparing the three pose-tracking algorithms

The three pose tracking algorithms had similar precision (CV) as depicted in Table 6. For example, for the eMOS of the front view walks in the top camera, the CV values were 0.48 (0.15), 0.42 (0.08), and 0.48 (0.14) for AlphaPose, OpenPose and Detectron, respectively, which indicates the similarity in precision between the three algorithms.

**Table 6:** comparing the precision (coefficient of variation) between the three pose tracking algorithms for top and eye-level cameras and in the front and back view walks

Gait variable	AlphaPose				OpenPose				Detectron			
	Front view walks		Back view walks		Front view walks		Back view walks		Front view walks		Back view walks	
	Top camera	Eye-level camera	Top camera	Eye-level camera	Top camera	Eye-level camera	Top camera	Eye-level camera	Top camera	Eye-level camera	Top camera	Eye-level camera
Cadence	0.09 (0.01)	0.08 (0.009)	0.09 (0.008)	0.09 (0.007)	0.08 (0.01)	0.08 (0.009)	0.09 (0.008)	0.09 (0.007)	0.09 (0.01)	0.08 (0.009)	0.09 (0.008)	0.09 (0.007)
Step time (s)	0.13 (0.10)	0.06 (0.02)	0.16 (0.18)	0.06 (0.03)	0.13 (0.10)	0.06 (0.02)	0.16 (0.18)	0.06 (0.03)	0.13 (0.10)	0.06 (0.02)	0.16 (0.18)	0.06 (0.03)
Step width	0.27 (0.08)	0.30 (0.07)	0.27 (0.08)	0.28 (0.07)	0.38 (0.21)	0.38 (0.12)	0.37 (0.10)	0.39 (0.09)	0.25 (0.07)	0.28 (0.06)	0.29 (0.12)	0.27 (0.08)
eMOS	0.48 (0.15)	0.43 (0.16)	0.41 (0.13)	0.38 (0.11)	0.42 (0.08)	0.46 (0.16)	0.43 (0.06)	0.44 (0.06)	0.48 (0.14)	0.39 (0.14)	0.37 (0.09)	0.43 (0.10)

**Fig. 4:**
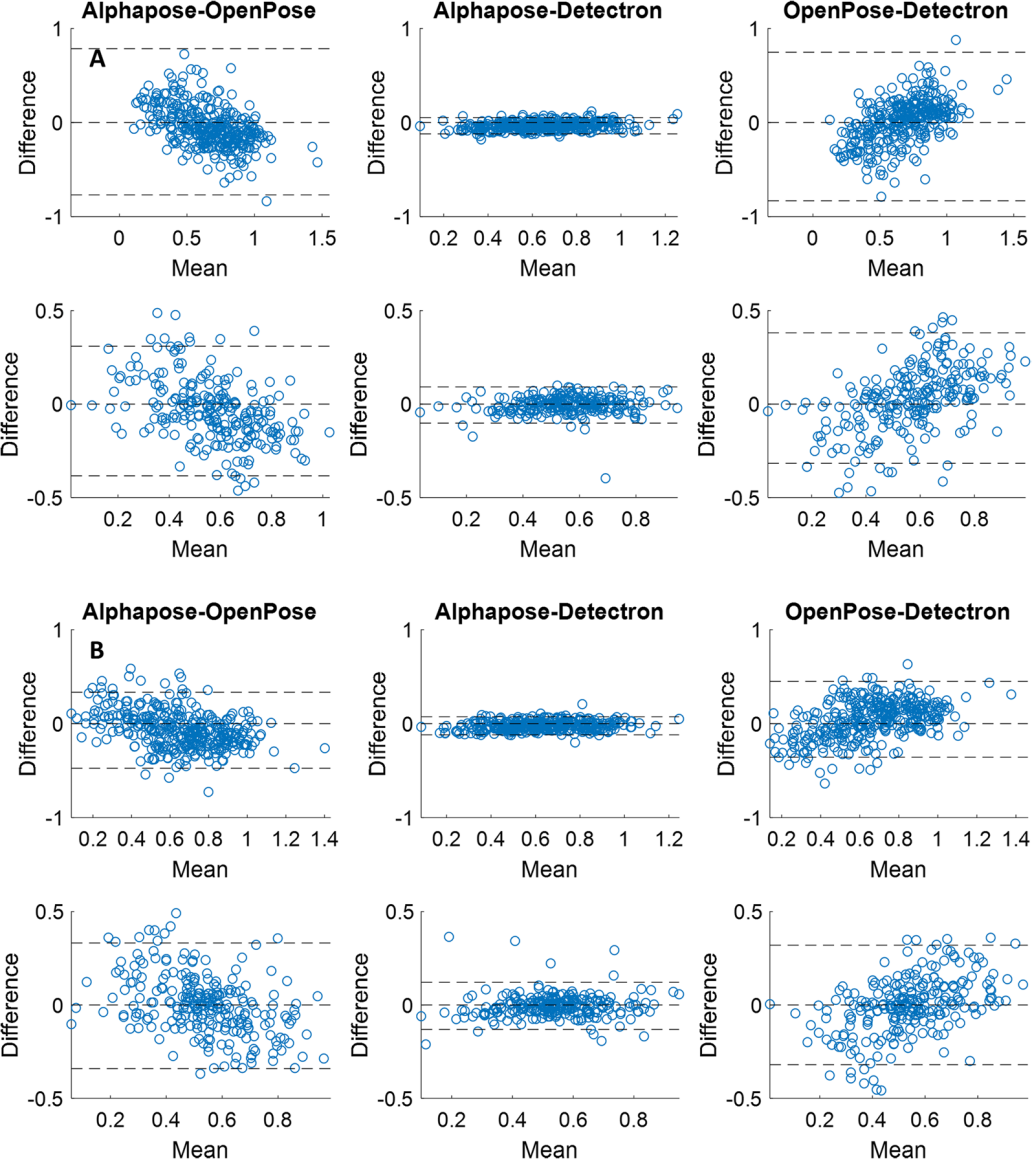
the Bland–Altman plots between pairs of the pose tracking algorithms for the step width from the videos of the top (**A**) and eye-level (**B**) cameras. The top row in each panel is for the front view walks and the bottom row is for the back view walks. The dashed lines are the lower and upper limits of agreement (1.96*standard deviation) as well as the zero line

**Fig. 5:**
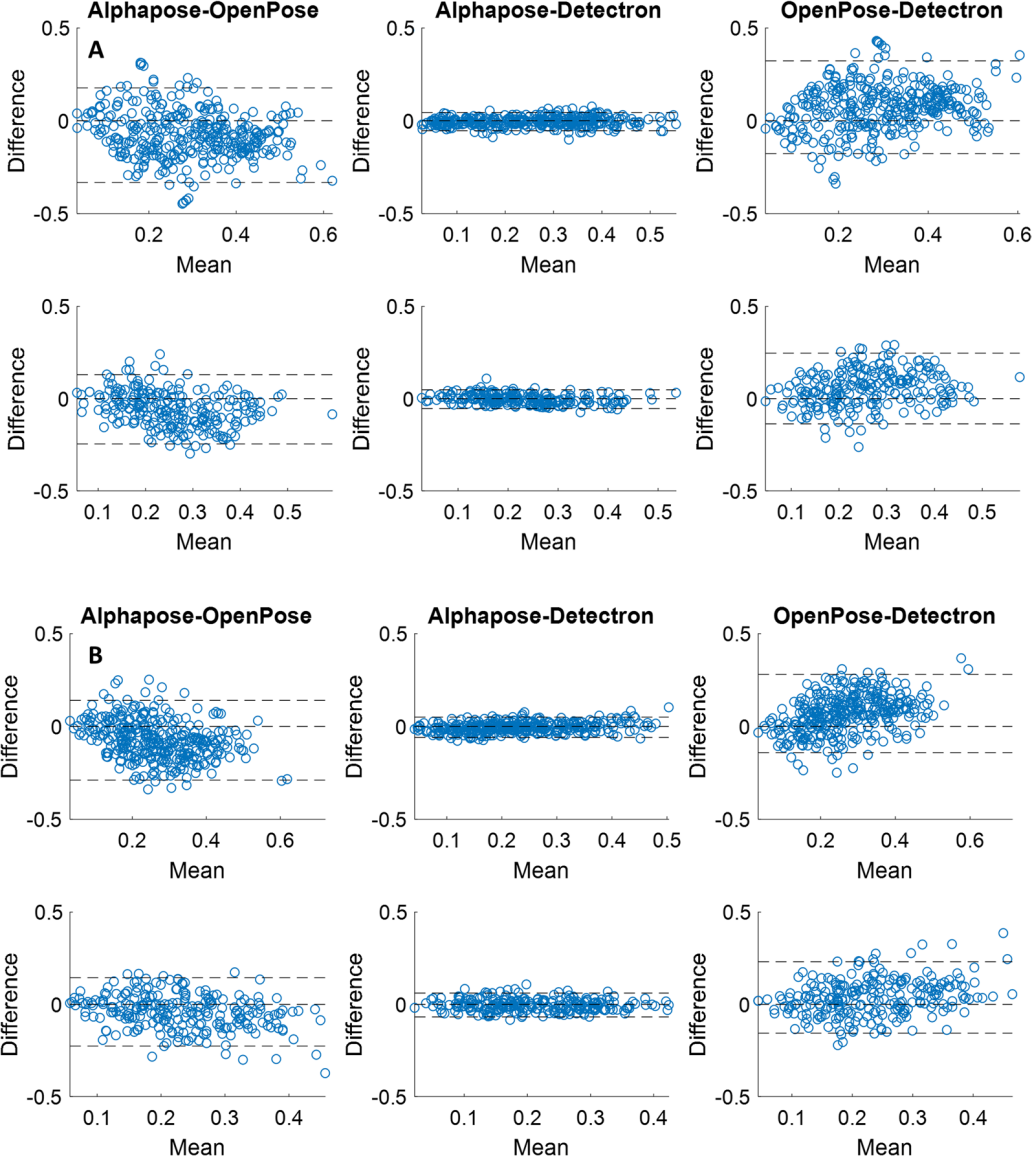
the Bland–Altman plots between pairs of the pose tracking algorithms for the estimated margin of stability (eMOS) from the videos of the top (**A**) and eye-level (**B**) cameras. The top row in each panel is for the front view walks and the bottom row is for the back view walks. The dashed lines are the lower and upper limits of agreement (1.96*standard deviation) as well as the zero line

The Bland–Altman plots for the three pairs of comparison between the pose trackers are presented in Figs. 4 and 5. Accordingly, for both step width (Fig. 4) and eMOS, there is more agreement between the AlphaPose and Detectron (middle columns) compared to between OpenPose and AlphaPose (left columns) or between OpenPose and Detectron (right columns). This is evident from the AlphaPose-Detectron pair’s narrower limits of agreement band (± 1.96*standard deviation) which contains 95% of the values.

## Discussion

We compared the gait variables calculated from 2D videos of standard RGB cameras using three pose tracking algorithms to those calculated from a 3D motion capture system. We performed the comparisons for videos of two cameras at two different heights, for front and back view walks, and when including gait variables at all steps of each participant or each participant’s averaged gait variables. Our findings revealed that, i) temporal (cadence and step time) calculated from the video pose tracking algorithms have a high correlation to that of 3D motion capture, but spatial (step width, eMOS) gait variables and gait variability (step time and width CV) are more weakly correlated, and ii) there are minimal differences between the three trackers evaluated, between the top and eye-level camera, and between the front and back view walks in terms of correlation of gait variables to the motion capture.

The standard RGB cameras have longer field of view compared to Microsoft Kinect (0.5 to 4.5 m) and this allows recording higher number of walking steps. Using standard RGB video cameras in this study, we were able to capture, on average, 14 and 12.3 steps per participant for each front and back view walking bouts, respectively. These numbers are significantly higher than that Microsoft Kinect camera can normally capture (3–6 steps) [3]. This highlights the importance of considering normal RGB cameras together with the pose tracking algorithms for quantitative gait tracking purposes as an alternative to Microsoft Kinect. It is nevertheless required to validate the pose tracking algorithms before using them in the clinical settings.

While previous studies on the validation of video pose tracking algorithms mainly used OpenPose and/or in sagittal view [9, 11, 12, 15], our study included three different pose trackers, videos from the front view, and in a sample population from older adults. We found that there is no difference between the three trackers in terms of the correlation of the gait variables to the 3D motion capture systems. This could be due to the similar training dataset these algorithms are pretrained on to identify body keypoints. That is, all these models were trained on the open-source Common Objects in Context (COCO) keypoint dataset [32]. Although there are technical differences in these algorithms (e.g. the bottom-up approach used in the OpenPose vs. the top-down approach in the Aplphapose and Detectron), it seems these differences had little effect on the quality of gait analysis in video pose tracking (see also Figs. 1 and 2). However, our findings indicated that among the three commonly used 2D pose trackers, there is more agreement between the gait variables calculated from AlphaPose and Detectron and less agreement between OpenPose and the other two algorithms (Figs. 4 and 5). While this finding does not mean that any of these three algorithms is more accurate (as we were not able to measure their accuracy against the IMU system), it can help future researchers with selecting the right pose trackers for their needs. It also remains to be investigated why OpenPose has less agreement with other two algorithms.

Our results also indicated that while the temporal gait variables of the video pose tracking have high correlation to the 3D motion capture, this was not the case for the spatial gait variables. Temporal gait variables are determined as the time difference between foot–ground contact and this requires relatively accurate determination of the foot contact moments. Thus, accurate estimation of temporal gait variables depends on identifying discrete events (e.g. peaks) in the time series of the segment and the relative time between these discrete events. Whether the value of these peaks in the time series accurately represent actual values is not important for calculating temporal gait variables. For the spatial gait variables, on the other hand, a more accurate localization of the body joints/segments is required. These findings are in line with the findings of [15]; when using OpenPose, they also found higher accuracy of temporal compared spatial gait measures in different camera views. Our results thus indicate that the three video pose tracking packages may not have enough accuracy in frame-by-frame tracking of walking videos. This could be because these packages are not specifically developed for quantitative gait monitoring purposes and are more for general gait pose estimation. This raises the need for developing video-pose tracking algorithms that are designed and trained for quantitative gait measurement purposes [33].

Consistent with the findings of [15], we also found that averaging gait variables over all steps of each participant improved the correlations although, the nonsignificant p-values of the correlations (except for cadence and step time and the back view eMOS of the eye-level camera) indicate that our sample size (n = 11) might not be enough for our conclusions. This improvement in correlation of the averaged values is understandable from a statistical point of view as individual gait variables have higher variance that results in lower correlation coefficients. This corresponds with the Robinson's paradox, which states that the correlation of aggregate quantities is not equal to the correlation of individual quantities [34]. This finding thus implies that the values these video pose tracking algorithms provide are more reliable at the average level compared to individual step level and thus provide an estimate of overall quality of the participant’s walking in a specific walking bout. The step-to-step values of the gait variables estimated from video pose tracking is thus less reliable and might not be used for clinical decision making.

There are a number of correlation values, mostly in the variability measures (i.e. step time CV and step width CV) where the correlation values are close to zero or even negative (Tables 3, 4,5). This is primarily due to low number of data points where each individual data point can contribute to the variance of dataset and thus have a strong effect on the correlation value. Note that to calculate variability, CV in this study, first the average of all data points of a walk (front or back view) of each participant is calculated that results in one value per participant. These findings imply that spatiotemporal variability measures cannot reliably be measured using the video pose tracking algorithms. However, consistent with other gait measures, this problem is lessened when calculating the variability measures over all walks of each participant (the “mean of all steps” in the Tables 3, 4 and 5) where the correlation values are increased.

There are several reasons why gait variables calculated from a 2D video pose tracking algorithm does not exactly match those calculated from a 3D motion capture. First, the body keypoints identified by pose tracking algorithms are not necessarily equivalent to the marker landmarks in the 3D motion capture systems. Second, the 2D video pose tracking algorithms could have parallax or perspective issues, which is not the case in the 3D motion capture systems [15]. Third, due to automatic focusing of video cameras, some frames could be blurred that negatively affects the capability of the pose tracking algorithms to identify body keypoints. Finally, in the back view walking videos, the trailing leg obscured the leading leg in a number of frames and thus leaving the pose tracking of the leading leg inaccurate. This, for example, caused inaccurate estimation of the foot contact time and position. For these reasons, it should not be expected that current video pose tracking algorithms have high correlations to the 3D motion capture systems.

Quantitative gait monitoring requires accurate estimates of the kinematics (i.e. position, velocity, and acceleration) of the joints and also accurate estimation of the gait events such as foot strike and foot off for its full potential. Current video pose tracking packages do not seem to prioritize measuring these quantities [33]. Thus, it seems reasonable to add kinematics as the labels to the training phase of the pose tracking algorithms. In addition, in most video pose tracking algorithms, sequential video frames are treated as independent frames (like static images) and the dynamics of the pose is ignored. A better approach could be treating the videos as dynamic images where the past and future frames of movement could be used to improve pose estimation in a given frame [33] and also to accurately detect gait events. Finally, human walking and movement, in general, are three-dimensional whereas most currently available pose tracking algorithms are two-dimensional. While there are attempts for 3D video pose tracking algorithms [35, 36], the accuracy and implementation of these algorithms for quantitative gait monitoring are still to be investigated [33].

One limitation of our study was that we tested only older adults who were able to walk independently for 20 m. Therefore, future studies should include older adults who have mobility problems and evaluate the feasibility of using vision-based systems for gait monitoring in these populations. In addition, we tested the performance of 2D pose estimation algorithms and the validity of the available 3D pose estimation packages is yet to be investigated, for example, in calculating joint angles in addition to gait measures. In addition, future studies should also consider assessing the validity of the pose tracking algorithms in more natural walking gaits where there are turns and stops. Finally, the sample size of 11 participants might be considered small, although it is in the range of studies on human walking. The initial plan was to recruit 20 older adults for this study. However, we had to stop data recording due to Covid-19 pandemic. Nevertheless, we were able to record a total of 373 and 241 steps for the front and back view walks, respectively, which is approximately equivalent to 34 and 22 steps per person for the front and back view walks, respectively.

## Conclusions

In this study, for the first time, we compared the validity of the three available pose tracking algorithms in a population of older adults in frontal view walking. Based on the findings of this study, we have the following recommendations for the use of 2D video pose tracking algorithms for gait monitoring purposes in older adults: (i) they should be mainly used for quantifying temporal gait variables, such as step time, and cadence, (ii) there are no major differences between top and eye-level camera viewing angles, and between the front and back view walks in terms of correlation of video gait variables to that of the motion capture, and (iii) these pose trackers provide better estimates of gait variables when averaged over all steps of each participant, and (iv) AlphaPose and Detectron showed more agreement, whereas OpenPose has a lower agreement with the other two algorithms. Future studies should engage in developing and validating video pose tracking algorithms that provide three-dimensional kinematics of body keypoints and are specifically designed and optimized for quantitative gait monitoring.

## Data Availability

The datasets used and/or analysed during the current study are available from the corresponding author on reasonable request.
